# Urban Rural Differences in Breast Cancer in New Zealand

**DOI:** 10.3390/ijerph13101000

**Published:** 2016-10-11

**Authors:** Ross Lawrenson, Chunhuan Lao, Mark Elwood, Charis Brown, Diana Sarfati, Ian Campbell

**Affiliations:** 1 National Institute of Demographic and Economic Analysis, The University of Waikato, Hamilton 3240, New Zealand; clao@waikato.ac.nz (C.L.); charis@waikato.ac.nz (C.B.); 2 School of Population Health, The University of Auckland, Auckland 1142, New Zealand; mark.elwood@auckland.ac.nz; 3 Department of Public Health, The University of Otago, Wellington 6021, New Zealand; diana.sarfati@otago.ac.nz; 4 School of Medicine, The University of Auckland, Auckland 1142, New Zealand; Ian.campbell@waikatodhb.health.nz

**Keywords:** rural, urban, breast cancer, Māori, equity, survival

## Abstract

Many rural communities have poor access to health services due to a combination of distance from specialist services and a relative shortage of general practitioners. Our aims were to compare the characteristics of urban and rural women with breast cancer in New Zealand, to assess breast cancer-specific and all-cause survival using the Kaplan–Meier method and Cox proportional hazards model, and to assess whether the impact of rurality is different for Māori and New Zealand (NZ) European women. We found that rural women tended to be older and were more likely to be Māori. Overall there were no differences between urban and rural women with regards their survival. Rural Māori tended to be older, more likely to be diagnosed with metastatic disease and less likely to be screen detected than urban Māori. Rural Māori women had inferior breast cancer-specific survival and all-cause survival at 10 years at 72.1% and 55.8% compared to 77.9% and 64.9% for urban Māori. The study shows that rather than being concerned that more needs to be done for rural women in general it is rural Māori women where we need to make extra efforts to ensure early stage at diagnosis and optimum treatment.

## 1. Introduction

Many rural communities have poor access to health services due to a combination of distance from specialist services and a relative shortage of general practitioners. These disadvantages can lead to differences in health outcomes. Breast cancer is the most commonly diagnosed cancer in New Zealand (NZ) women with 3000 new cases each year. It is also an important cause of death with 617 deaths per year [1]. Urban rural differences in breast cancer are well known. A comprehensive systematic review of the international literature suggested rural women were 19% more likely to be diagnosed with advanced stage at diagnosis [2]. Differences in mortality have also been shown between urban and rural women with breast cancer in Scotland [3], South Australia [4] and Germany [5]. It is difficult to generalise the findings from international studies as the definition of rurality can vary. For instance rural and remote communities in Australia and Canada will involve much greater distances from an urban medical centre than would be the case in Europe where population densities are greater.

In New Zealand, differences have been shown in the presentation and outcomes of breast cancer for Māori and Pacific women compared with NZ Europeans [6,7]. A prior study using NZ cancer registry data did not show differences in breast cancer mortality or stage of diagnosis for urban versus rural women after adjustment for age, ethnicity and socioeconomic status [8]. However the NZ cancer registry data has missing data and limited information on other characteristics that may influence outcomes, and the missing data may be differential by urban/rural status. For example, it may be that those living in more rural areas are more likely to have data missing. There is also variation in the definition of what is considered a rural population. This study is based on substantially more detailed clinical data available (including accurate staging, screening history and presence of biological markers) from two clinical breast cancer registers in the Waikato and Auckland regions [9]. Waikato District Health Board has a large rural community with 60% of patients living outside the main centre of Hamilton, while Auckland on the other hand is mainly an urban community. Our aims were (1) to compare the differences and characteristics of rural women particularly stage of diagnosis and tumour size as these are important measures of differences in diagnosis; (2) to assess breast cancer-specific and all-cause survival for urban and rural women with breast cancer; and (3) to assess whether the impact of rurality is different for Māori and NZ European women in New Zealand.

## 2. Methods

The population for this study included all women diagnosed with invasive breast cancer in the Waikato and Auckland regions from June 2000 to May 2013. The two registers have been collecting data prospectively on newly diagnosed women with breast cancer and have accurate data on demographic characteristics including place of domicile as well as clinical data on the breast cancer at diagnosis and subsequent treatment. Ethical approval for this study was obtained from the New Zealand Northern “A” Health and Disability Ethics Committee (Ref. No. 12/NTA/42).

The definition of urban and rural varies considerably across different studies and countries. Within NZ a number of different definitions have been considered [10,11]. For this study we have taken the seven different classifications of the urban rural profile classification basis of NZ statistics which is based on both domicile and work place. The seven categories are Major Urban; Satellite Urban; Independent Urban; Rural with high urban influence; Rural with moderate urban influence; Rural with low urban influence and Highly Rural/Remote. We have categorised rural areas with high urban influence to be urban as these communities generally are the high socioeconomic population living close to urban centres where these families work, shop and receive health care. On the other hand the NZ independent urban centres are those independent towns where general practitioners are classified as being rural and receive a rural bonus. Consequently we have redefined urban as either a major or satellite urban community or communities of high urban influence. Rural we have considered to be independent urban communities or those designated rural with moderate or low or no urban influence.

Other variables included in the study were: age (<40, 40–49, 50–59, 60–69, 70–79, 80+ years), ethnicity (NZ European, Māori, Pacific, Asian, others), cancer stage (I, II, III, IV), grade (1, 2, 3), tumour size (0–10, 10–20, 20–30, 30–50, 50+ mm), mode of detection (screen detected, not screen detected), and hormone status (Estrogen receptor (ER) and progesterone receptor (PR) negative, ER and/or PR positive). We first compared the characteristics between urban and rural women, and then compared urban and rural Māori and NZ European women. Key demographic and disease characteristics were described for the total cohort, for urban and rural populations and stratified by ethnicity.

Mortality information including date of death and cause of death were from both the combined registers and the Mortality Collection in New Zealand. For all-cause survival analyses, patients without mortality information were considered to be censored on the last updated date for Mortality Collection which was 31 December 2014. For cancer-specific analyses, deaths from other causes were censored on the date of death. Cancer-specific and all-cause survival were assessed for rural and urban women at 5 and 10 years using Kaplan–Meier method. Models were fitted using Cox proportional hazards models with cancer-specific and all-cause deaths as outcomes of interests, to compare outcomes for rural compared with urban women for the whole cohort and stratified by ethnicity. The proportional hazards assumption was tested for both breast cancer-specific and total mortality by creating a time-dependent covariate as an interaction term between urban-rural residence and survival time. The survival difference between subgroups was considered significant if the *p*-value was less than 0.05. All data analyses were performed in SPSS (IBM Corporation, New York, NY, USA).

## 3. Results

There were 12,372 women on the two registers with a diagnosis of invasive breast cancer. 2364 were classified as rural, living in an independent rural centre or a rural community, and 9885 were classified as urban (in 123 women domicile was not classified). When comparing the characteristics of urban and rural women with breast cancer, rural women tended to be older and were more likely to be Māori (Table 1). Stage of disease at diagnosis was similar and tumour size was also similar with 51.7% of rural women having a tumour size less than 20 mm and 50.3% of urban women (*p* = 0.238).

When we stratified by ethnicity, we find that rural Māori tended to be older, more likely to be diagnosed with metastatic breast cancer (*p* < 0.01) and less likely to be screen detected than urban Māori (Table 2). Rural NZ European were older and less likely to be diagnosed with Grade 3 cancer, but otherwise they had similar characteristics to urban NZ European including similar screen detected rates and similar stage at diagnosis.

When comparing 5-year and 10-year survival for urban versus rural women, we found that breast cancer survival is very similar for urban and rural women overall, but that for Māori women survival appears to be worse for rural compared with urban women (Table 3). Rural Māori women had inferior breast cancer-specific survival at 10 years at 72.1% compared to 77.9% for urban Māori (*p* = 0.072). The 5-year and 10-year all-cause survival was 71.6% and 55.8% for rural Māori women compared to 77.9% and 64.9% for urban Māori women (*p* = 0.017).

The hazard ratios for mortality using the Cox proportional hazards model (Table 4) showed that rural NZ European women have similar breast cancer-specific mortality and all-cause mortality compared to urban NZ European women after adjustment for age, cancer stage, tumour size, grade, hormonal status (ERPR), year of diagnosis, mode of detection and comorbidity. However, survival was poorer for rural Māori women compared with urban Māori women for both cancer-specific and all-cause survival. The unadjusted hazard ratio for breast cancer-specific mortality and the all-cause mortality for rural Māori compared with urban Māori was 1.31 (95% CI 0.97–1.76) and 1.33 (95% CI 1.05–1.68), respectively. The hazard ratio increased to 1.47 (95% CI 1.00–2.16) and 1.43 (95% CI 1.08–1.91), respectively, after adjustment for other factors. The proportionality assumption was tested for each of the 6 models shown in Table 4, with no significant departure from proportionality shown in all models except that of all rural versus all urban women for total mortality, where there was significant evidence of interaction between urban-rural residence and survival time on the hazard ratio (*p* = 0.029).

## 4. Discussion

The key findings from this study is that there is no evidence that rural women in New Zealand are more likely to present with advanced stage of disease or have poorer outcomes. In our two regions it appears that rural women with breast cancer are older than urban women and that there are proportionately more rural Māori than urban Māori. The cancer-specific mortality rates for rural women were similar suggesting that access to breast cancer treatment is at least as good for rural women as urban. 

However for Māori women with breast cancer outcomes are worse than for urban Māori. Rural Māori women do have lower rates of screen detected cancer and higher rates of metastatic disease. Overall there is a suggestion that both their breast cancer-specific mortality rate and all-cause mortality rate are not improved compared with urban Māori and may even be over 40% worse. One possible explanation for this is that deprivation may be more of an issue for Māori living in rural areas compared with non-Māori women which may have an additionally deleterious effect on survival. Similar observations have been reported from the USA. In one study socio-economic deprivation for native American Indians appeared to be more important than rurality [12] while in another study rural black American women were less likely to be screened and had more advanced disease at diagnosis [13]. Within New Zealand we know that there are health system barriers for Māori women with breast cancer. These include access to primary care and that when accessing specialist care lack of transport was a barrier—a key factor for rural women [14]. Access to primary care is important in that it has been shown that the more general practitioners per population, the earlier stage of diagnosis [15].

These findings are similar to those of our previous study using cancer registry data which showed no difference in stage or survival in rural women after adjustment for demographic factors. The screen detection rates in our rural women are re-assuring and suggest that our breast screening programs in Auckland and the Waikato are reaching rural women. The main concern is the overall lower rate in Māori women. These disparities are being addressed by Breast-screen Aoteoaroea. We have shown that rural Māori women can achieve high rates of breast screening if a concerted general practice based approach is taken to encouraging mammographic screening [16]. One of the factors that has been noted in Australia linked to poorer outcomes has been that rural women may be treated in a smaller centre by a low case load surgeon [17]. This is not a factor in our study as all women were treated in a high case load centre either in Auckland or Waikato. This may be one explanation for the lack of difference in overall outcomes.

The strengths of this study are that it is based on data from complete and high quality registers which have been collected prospectively. The weakness is that the study is dominated by the high number of urban women and relatively small numbers of rural Māori (311) which mean the results relating to Māori are less stable than the remainder of the results.

## 5. Conclusions

The study shows that, rather than being concerned that more needs to be done for rural women in general, it is rural Māori women where we need to make extra efforts to improve the later stage at diagnosis and ensure optimum treatment if we are to achieve equity in outcomes.

## Figures and Tables

**Table 1:** Characteristics of urban and rural women.

Characteristics	Rural		Urban		Total		*p*-Value (Chi-Square Test)
**Age Groups**							**<0.001**
<40	126	5.3%	669	6.8%	795	6.5%	
40–49	401	17.0%	2263	22.9%	2664	21.7%	
50–59	626	26.5%	2689	27.2%	3315	27.1%	
60–69	600	25.4%	2233	22.6%	2833	23.1%	
70–79	364	15.4%	1178	11.9%	1542	12.6%	
80+	247	10.4%	853	8.6%	1100	9.0%	
**Cancer Stage**							**0.066**
I	997	42.2%	4291	43.4%	5288	43.2%	
II	890	37.6%	3643	36.9%	4533	37.0%	
III	345	14.6%	1514	15.3%	1859	15.2%	
IV	132	5.6%	437	4.4%	569	4.6%	
**Grade**							**<0.001**
1	557	25.3%	2270	24.1%	2827	24.4%	
2	1113	50.5%	4281	45.5%	5394	46.5%	
3	535	24.3%	2850	30.3%	3385	29.2%	
Unknown	159		484		643		
**Tumour Size (mm)**							**0.238**
0–20	1123	51.7%	4726	50.3%	5849	50.5%	
20+	1050	48.3%	4674	49.7%	5724	49.5%	
Unknown	191		485		676		
**Mode of Detection**							**0.909**
Not screen detected	1458	61.7%	6084	61.5%	7542	61.6%	
Screen detected	906	38.3%	3801	38.5%	4707	38.4%	
**ERPR**							**0.034**
ER and PR negative	395	17.1%	1840	19.0%	2235	18.7%	
ER and/or PR positive	1910	82.9%	7819	81.0%	9729	81.3%	
Unknown	59		226		285		
**Ethnicity**							**<0.001**
NZ European	1932	82.2%	6943	71.2%	8875	73.3%	
Māori	311	13.2%	844	8.7%	1155	9.5%	
Pacific	38	1.6%	759	7.8%	797	6.6%	
Asian	47	2.0%	920	9.4%	967	8.0%	
Others	22	0.9%	285	2.9%	307	2.5%	
Unknown	14		134		148		
**Total**	2364	100%	9885	100%	12,249	100%	

ER: Estrogen receptor; RP: Progesterone receptor; NZ: New Zealand.

**Table 2:** Urban and rural women Māori and NZ European.

Characteristics	Māori						NZ European					
	Rural		Urban		Total		Rural		Urban		Total	
**Age Groups**												
***p*-Value (Chi-Square Test) 0.043**							**<0.001**					
<40	23	7.4%	74	8.8%	97	8.4%	95	4.9%	407	5.9%	502	5.7%
40–49	66	21.2%	238	28.2%	304	26.3%	303	15.7%	1391	20.0%	1694	19.1%
50–59	100	32.2%	264	31.3%	364	31.5%	488	25.3%	1796	25.9%	2284	25.7%
60–69	82	26.4%	183	21.7%	265	22.9%	491	25.4%	1650	23.8%	2141	24.1%
70–79	29	9.3%	72	8.5%	101	8.7%	324	16.8%	926	13.3%	1250	14.1%
80+	11	3.5%	13	1.5%	24	2.1%	231	12.0%	773	11.1%	1004	11.3%
**Cancer Stage**												
***p*-Value (Chi-Square Test) 0.076 **							**0.178**					
I	112	36.0%	315	37.3%	427	37.0%	837	43.3%	3136	45.2%	3973	44.8%
II	112	36.0%	313	37.1%	425	36.8%	726	37.6%	2546	36.7%	3272	36.9%
III	53	17.0%	162	19.2%	215	18.6%	279	14.4%	1003	14.4%	1282	14.4%
IV	34	10.9%	54	6.4%	88	7.6%	90	4.7%	258	3.7%	348	3.9%
**Grade**												
***p*-Value (Chi-Square Test) 0.065 **							**<0.001**					
1	56	19.7%	181	22.5%	237	21.7%	469	26.0%	1673	25.4%	2142	25.5%
2	158	55.6%	384	47.6%	542	49.7%	905	50.1%	3009	45.6%	3914	46.6%
3	70	24.6%	241	29.9%	311	28.5%	432	23.9%	1911	29.0%	2343	27.9%
Unknown	27		38		65		126		350		476	
**Tumour Size (mm)**												
***p*-Value (Chi-Square Test) 0.033 **							**0.949**					
0–20	130	48.0%	322	40.6%	452	42.4%	945	52.9%	3497	52.9%	4442	52.9%
20+	141	52.0%	472	59.4%	613	57.6%	843	47.1%	3109	47.1%	3952	47.1%
Unknown	40		50		90		144		337		481	
**Mode of detection**												
***p*-Value (Chi-Square Test) 0.323 **							**0.960**					
Not screen detected	208	66.9%	538	63.7%	746	64.6%	1170	60.6%	4209	60.6%	5379	60.6%
Screen detected	103	33.1%	306	36.3%	409	35.4%	762	39.4%	2734	39.4%	3496	39.4%
**ERPR**												
***p*-Value (Chi-Square Test) 0.389 **							**0.124**					
ER and PR negative	55	18.0%	131	15.8%	186	16.4%	324	17.2%	1275	18.8%	1599	18.4%
ER and/or PR positive	251	82.0%	696	84.2%	947	83.6%	1558	82.8%	5518	81.2%	7076	81.6%
Unknown	5		17		22		50		150		200	
**Total**	311	100%	844	100%	1155	100%	1932	100%	6943	100%	8875	100%

ER: Estrogen receptor; RP: Progesterone receptor; NZ: New Zealand.

**Table 3:** 5-year and 10-year breast cancer-specific survival and all-cause survival by Kaplan–Meier method.

Groups	5-Year (95% CI)		10-Year (95% CI)		Log Rank Test (*p*-Value)
**Breast cancer-specific survival**					
Rural women	86.6%	(85.1%–88.1%)	81.5%	(79.6%–83.4%)	0.211
Urban women	88.0%	(87.3%–88.7%)	82.5%	(81.5%–83.4%)	
Rural Māori women	78.3%	(73.2%–83.5%)	72.1%	(65.8%–78.5%)	0.072
Urban Māori women	84.1%	(81.4%–86.8%)	77.9%	(74.2%–81.5%)	
Rural European women	87.9%	(86.3%–89.4%)	82.7%	(80.7%–84.8%)	0.605
Urban European women	88.6%	(87.8%–89.4%)	83.0%	(81.9%–84.1%)	
**All-cause survival**					
Rural women	80.6%	(78.9%–82.3%)	67.8%	(65.4%–70.2%)	0.007
Urban women	82.7%	(81.9%–83.4%)	71.2%	(70.1%–72.4%)	
Rural Māori women	71.6%	(66.2%–77.0%)	55.8%	(48.2%–63.3%)	0.017
Urban Māori women	77.9%	(74.9%–80.9%)	64.9%	(60.6%–69.2%)	
Rural European women	81.9%	(80.0%–83.7%)	69.0%	(66.4%–71.6%)	0.394
Urban European women	82.0%	(81.1%–83.0%)	70.3%	(69.0%–71.7%)	

**Table 4:** Hazard ratio for mortality estimated by Cox proportional hazards model.

Groups	Unadjusted Hazard Ratio (95% CI)		Adjusted Hazard ^1^ Ratio (95% CI)	
**Breast cancer-specific mortality**				
Rural women compared with urban women	1.08	(0.96–1.22)	1.01	(0.87–1.17)
Rural Māori women compared with urban Māori women	1.31	(0.97–1.76)	1.47	(1.00–2.16)
Rural European women compared with urban European women	1.04	(0.90–1.19)	0.94	(0.79–1.10)
**All-cause mortality**				
Rural women compared with urban women	1.13	(1.03–1.24) *	1.04	(0.94–1.16)
Rural Māori women compared with urban Māori women	1.33	(1.05–1.68) *	1.43	(1.08–1.91) *
Rural European women compared with urban European women	1.05	(0.94–1.16)	0.90	(0.80–1.02)

^1^ Adjusted for age, (ethnicity), cancer stage, tumour size, grade, hormonal status (ERPR), year of diagnosis, mode of detection and comorbidity. * *p*-Value < 0.05.
